# P-2041. *In Vitro* Resistance Profiling of S-892216, a Second Generation SARS-CoV-2 3CL^pro^ Inhibitor for the Treatment of COVID-19

**DOI:** 10.1093/ofid/ofae631.2197

**Published:** 2025-01-29

**Authors:** Sho Kawashima, Haruaki Nobori, Miyako Okane, Takashi Hashimoto, Kae Inoue, Yuko Yamano, Keiko Funabiki, Michie Watanabe, Kaoru Baba, Keita Fukao, Teruhisa Kato

**Affiliations:** Shionogi & Co., Ltd., Toyonaka, Osaka, Japan; SHIONOGI & CO., LTD., Toyonaka-shi, Osaka, Japan; Shionogi TechnoAdvance Research CO., Ltd., Toyonaka, Osaka, Japan; Shionogi TechnoAdvance Research Co., Ltd., Toyonaka, Osaka, Japan; Shionogi TechnoAdvance Research Co., Ltd., Toyonaka, Osaka, Japan; Shionogi TechnoAdvance Research CO., Ltd., Toyonaka, Osaka, Japan; Shionogi TechnoAdvance Research CO., Ltd., Toyonaka, Osaka, Japan; Shionogi TechnoAdvance Research CO., Ltd., Toyonaka, Osaka, Japan; Shionogi TechnoAdvance Research, Co., Ltd., Toyonaka, Osaka, Japan; Shionogi&Co., Ltd., Toyonaka, Osaka, Japan; Shionogi & Co., Ltd., Toyonaka, Osaka, Japan

## Abstract

**Background:**

S-892216 is a candidate for the treatment of COVID-19 that has 3CL^pro^ inhibitory activity and antiviral activity. In this study, the susceptibility of S-892216 was evaluated against SARS-CoV-2 with 3CL^pro^ substitution associated with reduced susceptibility to other 3CL^pro^ inhibitors, ensitrelvir and nirmatrelvir. Additionally, in vitro selection of SARS-CoV-2 with reduced susceptibility to S-892216 was conducted.

**Methods:**

Reverse genetics-derived SARS-CoV-2 (rgSARS-CoV-2) with 3CL^pro^ substitutions including M49L and E166V were generated using circular polymerase extension reaction (CPER) system The susceptibility of S-892216 was assessed using rgSARS-CoV-2 infected VeroE6/TMPRSS2 cells. For the selection of SARS-CoV-2 with reduced susceptibility to S-892216 in vitro, VeroE6/TMPRSS2 cells infected with SARS-CoV-2 Omicron BE.1/BA.5-like variant (hCoV-19/Japan/TY41-702/2022) were cultured in the presence of S-892216 and passaged 10 times. Genotyping analysis of 3CL^pro^ and its cleavage sites in the isolated SARS-CoV-2 were then conducted. The susceptibility of S-892216 and nirmatrelvir against the isolated viruses were assessed.

**Results:**

Fold change (FC) values of S-892216 against rgSARS-CoV-2 with 3CL^pro^ substitutions M49L and E166V were < 2-fold. SARS-CoV-2 with 3CL^pro^ substitutions of D48E, M49K, L50F, N221K, and P252L were isolated from the in vitro virus selection. The FC values of each compound against the isolated SARS-CoV-2 were 3.51- to 28.7-fold for S-892216 and 0.736- to 3.25- fold for nirmatrelvir.

**Conclusion:**

The isolated SARS-CoV-2- strains with reduced susceptibility to S-892216 carried 3CL^pro^ mutation - indicating that the target site is 3CLpro, did not show cross-resistance to nirmatrelvir. Furthermore, S-892216 did not show cross-resistance against nirmatrelvir and ensitrelvir-reduced susceptibility strains. These data suggest S-892216 might be effective if described ensitrelvir- and nirmatrelvir-resistant SARS-CoV-2 variants become relevant in the clinical setting.

**Disclosures:**

Sho Kawashima, Shionogi & Co., Ltd.: Employee Haruaki Nobori, PhD, Shionogi & Co., Ltd.: Employee|Shionogi & Co., Ltd.: Stocks/Bonds (Private Company) Miyako Okane, n/a, Shionogi & Co., Ltd.: Stocks/Bonds (Private Company)|Shionogi TechnoAdvance Research CO., Ltd.: employee Takashi Hashimoto, n/a, Shionogi TechnoAdvance Research CO., Ltd.: employee Kae Inoue, n/a, Shionogi & Co., Ltd.: Stocks/Bonds (Private Company)|Shionogi TechnoAdvance Research CO., Ltd.: employee Yuko Yamano, n/a, Shionogi TechnoAdvance Research CO., Ltd.: employee Keiko Funabiki, n/a, Shionogi TechnoAdvance Research CO., Ltd.: ex-employee Michie Watanabe, n/a, Shionogi TechnoAdvance Research CO., Ltd.: employee Kaoru Baba, n/a, Shionogi TechnoAdvance Research: Employee Keita Fukao, MD, SHIONOGI and CO., LTD.: Stocks/Bonds (Private Company) Teruhisa Kato, PhD, Shionogi & Co., Ltd.: employee|Shionogi & Co., Ltd.: Stocks/Bonds (Private Company)

